# Long-Lasting Anti-Inflammatory Activity of Human Microfragmented Adipose Tissue

**DOI:** 10.1155/2019/5901479

**Published:** 2019-02-19

**Authors:** Sara Nava, Valeria Sordi, Luisa Pascucci, Carlo Tremolada, Emilio Ciusani, Offer Zeira, Moris Cadei, Gianni Soldati, Augusto Pessina, Eugenio Parati, Mark Slevin, Giulio Alessandri

**Affiliations:** ^1^Cellular Neurobiology Laboratory, Department of Cerebrovascular Diseases, Fondazione IRCCS Neurological Institute C. Besta, Milan, Italy; ^2^Diabetes Research Institute, IRCCS San Raffaele Scientific Institute, 20132 Milan, Italy; ^3^Department of Veterinary Medicine, University of Perugia, Perugia, Italy; ^4^Image Institute, Milan, Italy; ^5^School of Healthcare Science, John Dalton Building, Manchester Metropolitan University, Chester Street, Manchester M1 5GD, UK; ^6^Laboratory of Clinical Pathology and Neurogenetic Medicine, Fondazione IRCCS Neurological Institute C. Besta, Milan, Italy; ^7^San Michele Veterinary Hospital, Tavezzano con Villavesco, Lodi, Italy; ^8^Section of Pathological Anatomy DMMT, University of Brescia, Brescia, Italy; ^9^Swiss Stem Cell Foundation, In Pasquée 23, Gentilino, CH-6925 Lugano, Switzerland; ^10^Department of Biomedical, Surgical and Dental Sciences, University of Milan, Milan, Italy; ^11^Weifang Medical University, Weifang, China; ^12^University of Medicine and Pharmacy, Targu Mures, Romania

## Abstract

Over the last few years, human microfragmented adipose tissue (MFAT), containing significant levels of mesenchymal stromal cells (MSCs) and obtained from fat lipoaspirate (LP) through a minimal manipulation in a closed system device, has been successfully used in aesthetic medicine as well as in orthopedic and general surgery. Interestingly, in orthopedic diseases, this ready-to-use adipose tissue cell derivative seems to have a prolonged time efficacy even upon a single shot injection into osteoarthritic tissues. Here, we investigated the long-term survival and content of MSCs as well the anti-inflammatory activity of LP and its derived MFAT in vitro, with the aim to better understand a possible in vivo mechanism of action. MFAT and LP specimens from 17 human donors were investigated side by side. During a long-term culture in serum-free medium, we found that the total cell number as well the MSC content in MFAT decreased more slowly if compared to those from LP specimens. The analysis of cytokines and growth factors secreted into the conditioned medium (CM) was similar in MFAT and LP during the first week of culture, but the total amount of cytokines secreted by LP decreased much more rapidly than those produced by MFAT during prolonged culture (up to 28 days). Similarly, the addition of MFAT-CM recovered at early (3-7 days) and late stage (14-28 days) of culture strongly inhibited inflammatory function of U937 monocyte cell line, whereas the anti-inflammatory activity of LP-CM was drastically reduced after only 7 days of culture. We conclude that MFAT is an effective preparation with a long-lasting anti-inflammatory activity probably mediated by a long-term survival of their MSC content that releases a combination of cytokines that affect several mechanisms involved in inflammation processes.

## 1. Introduction

Autologous use of adipose mesenchymal stem/stromal cells (MSCs), or the stromal vascular fraction (SVF) isolated from liposuction of fat tissue, has slowly gained support for the treatment of a variety of pathological conditions from osteoarthritis through skin wound healing to stroke and brain injury [1]. With very few or none apparent side effects and a potential tissue regenerative capacity, these fat-derived “bioreactors” could hold the key to next-generation therapies being more effective in recreation of like-for-like three-dimensional tissue repair. SVF can act as a three-dimensional matrix or scaffold containing activated cellular components including adipocytes, pericytes/pericyte-derived MSCs, and potentially “angiogenic” endothelial cells (ECs) [2, 3]. To date, a detailed understanding of the mechanisms through which these biological materials are able to moderate tissue repair is required to work hand in hand with an appreciation of the safety of such therapies.

In particular, the adipose MSC component of these SVFs has been highlighted in most detail, undergoing consideration for treatment of osteoarthritis and cartilage repair [4, 5], anti-inflammatory stroke therapy, and treatment for Parkinson's disease [6, 7]. In addition, it has shown promise for the treatment of musculoskeletal regeneration [8] and treatment of complex anal fistula [9].

The anti-inflammatory and cell protective properties of the fat tissue are of great interest, in particular the MSC secretome which contains specific anti-inflammatory and immunosuppressive cytokines and growth factors including iNOS, IDO, PGE_2_, TSG6, HO1, TGF-*β*, and galectins [10–12], but also contains extracellular vesicles (EVs) which recapitulate some MSC functions. Importantly, MSC-derived EVs have been shown to retain regenerative and anti-inflammatory properties and thus proposed to be used as cell-free therapies [13, 14]. The specific microenvironment within inflammatory tissue dictates MSC response and ultimately phenotypical variations; therefore, it is critical to understand MSC homing and secretion in order to postulate possible therapeutic applications [10, 15].

While several mechanical and enzymatic protocols have been used to prepare fat MSCs or the most impure SVF, involving centrifugation, washing, and filtration [2], most recently, Tremolada and colleagues [16] developed a relatively simple self-contained mechanical technique to create a microfragmented adipose-derived fraction (MFAT) through an enzyme-free technology, able to convert lipoaspirate (LP) into MFAT using a device named Lipogems®. This technique reduces the size of the adipose tissue clusters by means of mild mechanical forces and eliminates oil and blood residue. The technique is gentle and provides microfragmented fat in a short time (15-20 min), without expansion and/or enzymatic treatment. Through this technology, it was demonstrated that MFAT contains a significant number of MSCs that can be directly injected into patients [17]. This nonexpanded MFAT has been shown to possess regenerative properties, particularly when injected into inflammatory or ischemic tissues [16, 18]; recently, it has been successfully used in aesthetic medicine as well as in orthopedic diseases. Interestingly, in orthopedic diseases, this ready-to-use adipose tissue cell derivative has shown a very prolonged time efficacy even upon a single shot injection into dogs with osteoarthritic disease [18]. Our group has shown that this biomaterial could block the proinflammatory activities of U937 macrophages/monocyte cell line by reducing their ability to bind activate ECs [19] while intraperitoneal injection of MFAT significantly attenuated inflammation following caecal ligation in a mouse model of sepsis [18]. Based on these experimental and clinical results, in this work, we aimed to identify the mechanistic detail differentiating MFAT from the standard LP. More specifically, we investigated the long-term survival and content of MSCs as well the anti-inflammatory activity of LP and its derived MFAT. We also analyzed their secretome in vitro. We found that MFAT specimens, cultured under serum-free conditions, contained a significant amount of MSCs and have an impressive capacity to secrete molecules with anti-inflammatory properties whose activity lasts for weeks; vice versa, MSC content and secretome activity of LP counterpart, under the same culture conditions, decay rapidly (within a week).

## 2. Materials and Methods

### 2.1. MFAT Processing from Lipoaspirate

According to the policies approved by the Institutional Review Boards for Human Studies local ethical committees (IRB 48/2013, Istituto Neurologico Carlo Besta), 17 different fat donors were investigated. In this study, LPs were obtained from patients undergoing plastic surgery; written informed consent was obtained from all donors. MFAT specimens were prepared from LP counterpart of the same donor, as previously described [17]. Briefly, 100 ml of LP was obtained from each patient, and 50 ml of LP was used for MFAT preparation by using a standard 225 ml Lipogems® device (provided by Lipogems® International, Milan, Italy). The LP collected by syringe is pushed into Lipogems® device through a filter for a first cluster reduction; afterwards, the five stainless steel marbles inside the device are shaken to disaggregate fat material producing cell clusters and microfragmented fat tissue that migrated to the top of device, while blood contaminating cells and undesired fat residues are removed by a gravity counterflow of saline solution. When the solution inside the device appears yellow and clear, the device was turned upside down and a second microfragmentation of the tissue was obtained by pushing the adipose clusters with a syringe through a size reduction filter. At the end of this procedure, MFAT product was aspirated by a syringe connected with the device and was ready for investigation.

### 2.2. Preparation of Conditioned Medium (CM) from MFAT (MFAT-CM), LP (LP-CM), and Their Isolated MSCs (MSCs-CM)

**Figure 1:**
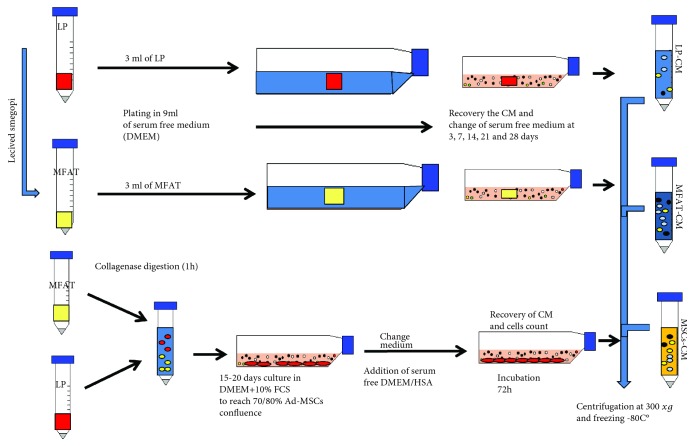
Schematic procedure for the preparation of conditioned medium (CM) from MFAT (MFAT-CM), LP (LP-CM), and Ad-MSCs (Ad-MSCs-CM). Specimens of LP and its counterpart MFAT (3 ml each) were washed in PBS three times and seeded in T75 flask in 9 ml of plain DMEM (without exogenous proteins implementation). At each incubation time, the medium was aspirated and replaced with an equal amount of fresh DMEM. At the end of incubation, all the CMs were centrifuged, filtered (0.2 *μ*m), and frozen (-80°C) until used. To obtain MSCs-CM from either MFAT or LP, tissues (3 ml) were digested with collagenase. The cell pellets were cultured for 15-20 days in DMEM + 10% FCS. When cells reached 80% of confluence, medium was substituted with an equal volume of DMEM + 0.2%HSA. After 72 h, the MSCs-CM was recovered and the cells were harvested and counted. The MSCs-CM was processed as described above.

Specimens of LP and its counterpart MFAT, freshly obtained from patients, were washed in PBS three times by centrifugation at 300 ×*g* for 10 min. After discarding PBS, 3 ml of MFAT and LP was seeded in T75 flask in 9 ml of DMEM (Gibco, Life Technologies, Monza, Italy) serum-free plain medium. The flasks were incubated for 3, 7, 14, 21, and 28 days at 37°C in 5% CO_2_. At the end of each incubation time, the conditioned medium (CM) was recovered and equal amount of fresh medium was added. MFAT-CM and LP-CM were centrifuged at 300 ×*g* for 10 min, filtered 0.22 *μ*m, aliquoted, and stored at -80°C until use. MSCs from 3 ml MFAT or LP were isolated after collagenase (0.25% *w*/*v*, Sigma, St. Louis, MO, USA) digestion as previously described [19]. The MSCs were cultured in DMEM + 10% FCS (Gibco, Dublin, Ireland) until reaching 70-80% of confluence, and then the cells were detached with trypsin, counted, and seeded at 1 × 10^6^ in T75 flask in 9 ml of DMEM + 0.2% human serum albumin (HAS, Baxalta Innovations GmbH, Vienna, Austria). The MSCs-CM was prepared upon incubation at 37°C for 3 days. Prolonged incubation was not performed due to MSC apoptosis under serum-free medium culture condition. MSCs-CM was centrifuged, filtered, and stored at -80°C until used. The schematic preparation of CM from MFAT, LP, and MSCs is also reported in Figure 1.

### 2.3. Quantification of Protein Content of MFAT and LP Specimens

To quantify the protein content in MFAT and LP, 1.5 ml of both specimens for each donor was used. Briefly, fresh tissues, after three washes in PBS by centrifugation (300 ×*g*, 10 min), were kept ice and sonicated in PBS (without Ca and Mg) and 500 *μ*l of protease inhibitors (Sigma, Italy). The tissue were then centrifuged at 27,000 ×*g* at 4°C for 20 min. The supernatants were recovered and transferred in a new tube and analyzed for protein content by the Lowry method [20].

### 2.4. Quantification of Cells and DNA Content in MFAT and LP Specimens

3 ml of MFAT and LP specimens was used to evaluate cells and DNA content. After overnight collagenase digestion, all the cells derived from MFAT and LP were washed twice in PBS. Half of the final cell was then frozen and used for genomic DNA extraction using the QIAamp DNA mini kit following the manufacturer's instructions and resuspended in 50 *μ*l of appropriate buffer (QIAGEN, Italy). In order to quantitate the approximate number of cell in each MFAT and LP sample, we followed two procedures: (1) each cell pellet was resuspended in PBS, filtered through 40 *μ*m pore size to remove undigested aggregates, and then centrifuged (300 ×*g*, 10 min) and resuspended in trypan blue solution and finally counted by hemocytometer and (2) DNA was extracted by cell pellet of a given number of peripheral blood mononuclear cells (PBMNCs) obtained by density gradient centrifugation on Ficoll-Hypaque (Sigma, Italy) from an healthy volunteer. DNA concentration in each sample was evaluated by absorbance at 260 nm using a NanoDrop microvolume spectrophotometer (Thermo Fisher, Italy). DNA samples derived by PBMNC pellets were used to draw a calibration curve by which an approximate number of cell in each pellet was calculated.

### 2.5. Characterization and Quantification of MSCs and CD31^+^/ECs in MFAT and LP Specimens

To quantify the MSCs (CD31^−^) and ECs (CD31^+^) from fresh and cultured MFAT and LP specimens, 3 to 5 ml of fat samples was used. MFAT and LP specimens were cultured in DMEM serum-free medium and at days 0, 7, 14, 21, and 28 digested with collagenase to evaluate the total cells and MSC content. After collagenase digestion, the obtained cell pellets were filtered through 40 *μ*m pore size and processed for CD31^+^ selection by using magnetic microbeads (Invitrogen, Italy, CELLection™ Pan Mouse IgG Kit,) as previously described [19]. CD31^+^ and CD31^−^ cells were analyzed for endothelial and mesenchymal markers, respectively, by flow cytometry. Briefly, cells were resuspended in PBS at a concentration of 1 × 10^5^/100 *μ*l and incubated with 10 *μ*l of conjugated primary antibody for 30 min at 4°C in the dark. Phycoerythrin (PE) conjugate antibodies were used: anti-human CD34 (BD Pharmingen™, San Jose, CA, USA; working dilution 1 : 10) for CD31^+^ selected cells, anti-human CD90 (Millipore, Billerica, MA, USA; working dilution 1 : 10), and anti-human CD105 and anti-human CD73 (BD Pharmingen™, working dilution 1 : 10) for CD31 cells. Unspecific staining was determined with appropriate isotype controls. At least 20,000 events were acquired for each sample on a FACS Advantage SE (BD Biosciences, San Diego, CA, USA) flow cytometer, and the acquisition analyses were performed using a CellQuest software (BD Biosciences). CD31 cells were also investigated for mesenchymal markers by immunocytochemical analysis through cytoinclusion technique [21]. Briefly, cell pellets were resuspended in 40 *μ*l of Matrigel (BD Biosciences, Franklin Lakes, NJ, USA) and left to jellify for one hour at 37°C. The samples were then placed in plastic boxes and fixed in 10% formalin. Cells were analyzed for the expression of CD90, CD105, and CD73 (BD Biosciences, Franklin Lakes, NJ, USA).

### 2.6. Analysis of Secretome of MFAT and LP Specimens

Human cytokines/chemokines were detected using multiplex bead assays based on xMAP technology (Bio-Plex Human Cytokine 27-Plex Panel; Bio-Plex Human Group II Cytokine 23-Plex Panel; Bio-Rad Laboratories, Hercules, CA, USA). The CM from MSCs was collected after 3 days of culture while the CM from MFAT and LP was collected at 3, 7, 14, 21, and 28 days. All CMs were assayed for a total of 48 proteins: IL-1b, IL-2, IL-4, IL-6, IL-7, CXCL8, IL-10, IL-12 (p70), IL-13, IL-15, IL-17, CCL11, *β*-FGF, G-CSF, GM-CSF, IFN-*γ*, CXCL10, CCL2, CCL3, CCL4, PDGF-BB, CCL5, TNF-*α*, VEGF, IL-1*α*, IL-3, IL-12 (p40), IL-16, IL-18, CCL27, CXCL1, HGF, IFN-*α*2, LIF, CCL7, M-CSF, MIF, CXCL9, *β*-NGF, SCF, SCGF-*β*, CXCL12, TNF-*β*, and TRAIL.

### 2.7. Evaluation of Anti-Inflammatory Activity of MFAT and LP

The anti-inflammatory activity of MFAT-CM and LP-CM recovered at different incubation time was tested on the U937 monocyte/macrophage cell line (ATCC, Manassas, VA, USA). These cells were routinely maintained in RPMI media implemented with 10% FBS and expanded twice a week. Corning Costar Transwell 5 *μ*m pore size (Celbio, Milan, Italy) supports were used to test the effect of MFAT-CM and LP-CM on U937 migration. MCP-1 chemokine (10 ng/ml, Sigma-Aldrich, St. Louis, MO, USA) was used as positive chemotactic factor. For each test, 2 × 10^5^cells in 200 *μ*l of DMEM + 0.2% BSA were placed on the top of the membrane insert. To evaluate spontaneous migration, 500 *μ*l of control DMEM + 0.2% BSA medium was added to the lower compartment of the wells. To evaluate MFAT-CM and LP-CM activities, different dilutions were added in the lower compartment of each well in the presence or in the absence of MCP-1. Migration assay was carried out for 6 h at 37°C in 5% CO_2_, and then the membrane inserts were removed, fixed in 10% formalin, and stained with Wright's solution. Cells attached to the upper surface of the filter were removed with a swab, and cells migrated across the membrane were counted by microscopically examining the lower surface. Reported data represent the total number of cells found in 10 different fields for each membrane at 40x magnification. Each determination was done in duplicate. ELISA kits were used to quantify the production of RANTES and MCP-1 (R&D Systems, UK, Europe) by U937 cells line under basal culture conditions, in the presence of inflammatory stimuli (LPS 1 *μ*g/ml, Sigma, Italy) combined or not with different dilutions of MFAT-CM and LP-CM. All the data were normalized for 10^6^ U937 in 24 h of incubation subtracting the basal level of the same chemokines present in the MFAT-CM and in LP-CM.

## 3. Results

### 3.1. Characterization of MFAT and LP Samples

LP was obtained from 17 human donors; half volume of each LP specimen was processed by Lipogems® device to obtain the corresponding MFAT; LP and MFAT of each donor were characterized for protein concentration, DNA content, the total number of cells obtained after collagenase digestion, CD31^+^ % cells to estimate the number of ECs, and finally the number of MSCs by evaluating the positive cell expression for CD105^+^, CD90^+^, and CD73^+^ [22].

**Figure 2:**
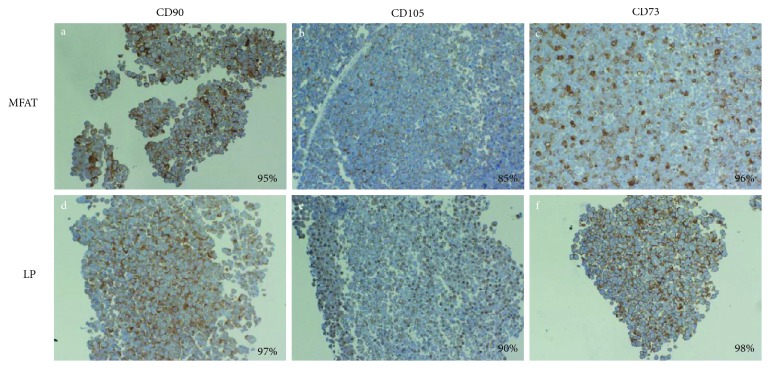
Expression of CD90, CD105, and CD73 markers in CD31 cells derived from MFAT and LP specimens. CD31 cells isolated from MFAT and LP specimens were cultured for 14 days. At the end of incubation, the cells were recovered, cytoincluded in Matrigel, and analyzed by immuocytochemistry. The figures (10x magnification) shows MFAT (a, b, and c) and LP (d, e, and f) staining for CD90, CD105, and CD73, respectively. The % of positive cells is reported at the lower right corner of each picture.

All the results are summarized in Table 1. We observed a significant variability among donors. However, the total protein concentrations, DNA content, and the total number of cells were higher in LP compared to MFAT. Vice versa, the % of cells positive for CD31, an endothelial marker, was higher in MFAT. Interestingly, the total absolute number of MSCs contained in LP was superior than those in MFAT, but the % of MSCs in the total number of CD31 cells was lower in LP (median value 16.6%) than in MFAT (median value 26.9%). Therefore, this data confirms previous reports showing that in MFAT, MSCs, and ECs are more concentrated than in LP [17]. To further confirm the presence of MSC phenotype in the selected CD31 cell population of MFAT and LP, CD31 cells were cultured in DMEM + 10% FCS for 2 weeks and then stained by immunocytochemistry for CD105, CD90, and CD73 mesenchymal markers. A very high expression of all markers (up to 90% positivity) was found in CD31 cell population of both MFAT and LP (Figure 2). The clonogenicity and the differentiation potential of LP and MFAT cells were confirmed in a previous published paper ([23], data not shown).

### 3.2. Analysis of Secretome from LP and MFAT Specimens

To analyze the secretome derived from MFAT and LP specimens as well as from their isolated MSC cultures, we used a procedure schematically reported in Figure 1. Briefly, MFAT-CM and LP-CM were obtained by seeding an equal volume (3 ml) of MFAT and LP specimens in 9 ml of DMEM serum-free medium for different incubation time (from 72 h to 28 days). The CM from MSCs (MSCs-CM) was analyzed only at 72 h of incubation because prolonged time of MSC (both isolated from MFAT and LP) incubation under serum-free culture condition induced strong cell apoptosis and mortality.  Table 2 reports the secretome analysis of MSCs-CM, MFAT-CM, and LP-CM at 72 h of incubation. In all the CM, a very similar and significant amount of cytokines and growth factors was found. However, the CM from isolated MSCs derived from MFAT contained higher amount of IL6 and MCP-1 cytokines as well as VEGF and SCGF-*β* growth factors when compared to MFAT-CM or LP-CM. Similar results were obtained with MSCs derived from LP (data not shown). On the contrary, MFAT-CM and LP-CM secreted higher level of *β*-FGF and HGF growth factors and IL8, IL16, MIG, and MIF cytokines respect to MSCs.

Interestingly, the intense secretory activity of MFAT and LP during the first 72 h of incubation was very similar in terms of quality and quantity for cytokine secretion. Only G-SCF was significantly higher in MFAT-CM compared to LP-CM, suggesting that the procedure of microfragmentation of LP to produce MFAT did not alter the releasing pathway of cells.

In order to evaluate the secretory activity of MFAT and LP during prolonged incubation time, the analysis of MFAT-CM and LP-CM was repeated at 7, 14, and 28 days (Table 3). After 7 days of incubation, in LP-CM, we found a higher level of cytokines if compared to MFAT-CM (32,872 ± 9854 vs. 20,039 ± 4387 pg/ml). However, the analysis of CM at 14 days demonstrated a dramatic and rapid decline of cytokines and growth factor secretion by LP respect to MFAT-CM, in which the quantity of proteins remained stable. After 28 days of culture, the differences between MFAT and LP secretome were even more evident: MFAT continues to release a significant amount of cytokines (23,057 ± 6590 pg/ml); on the contrary, the level of cytokines secreted by LP was dramatically reduced (706 ± 154 pg/ml).

### 3.3. Long-Lasting Survival of MSCs in MFAT Specimens

**Figure 3:**
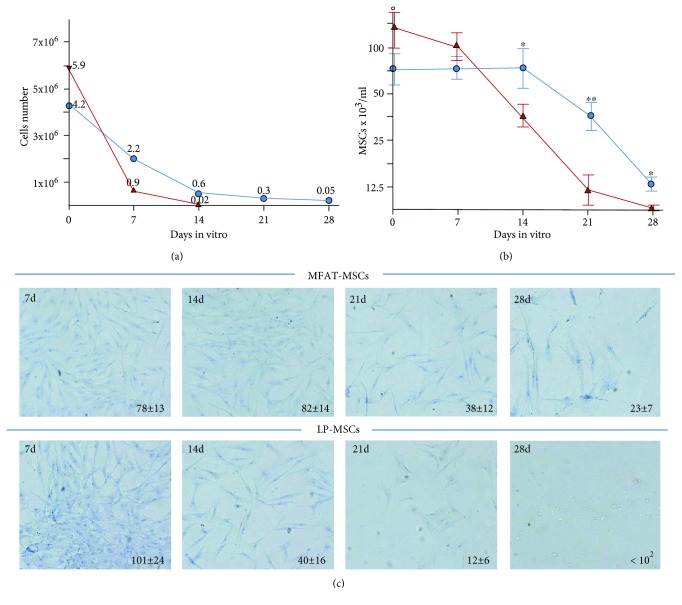
Total cell number and MSC content in LP and MFAT specimens. An identical volume (3 ml) of MFAT (blue line) and LP (red line) specimens was digested with collagenase upon cultivation in DMEM plain medium for 0, 7, 14, 21, and 28 days to evaluate the total cells (a) and MSC content (b). On day 0, the total cells/ml as well the MSC content were higher in LP than in MFAT (total cells LP = 6.3 ± 4.4 × 10^5^ vs. MFAT = 3.7 ± 1.8 × 10^5^; MSCs LP 14.9 ± 6.3 × 10^3^ vs. MFAT 10.1 ± 5.8 × 10^3^). After 14 days of culture, both total cells and MSCs were significantly reduced in LP, whereas in MFAT remained stable. (c) Pictures (20x magnification) of MSCs isolated and seeded in T25 flask, from LP and MFAT specimens. At the lower right corner of the pictures, the cell number/field is reported and represent the average ± SD of 5 different fields. Eight different donors were analyzed.

MFAT and LP specimens were cultured in DMEM serum-free medium and at days 0, 7, 14, 21, and 28 digested with collagenase to evaluate the total cells and MSC content (Figure 3). The total cell number and viability resulted reduced during culture in a more evident way for LP (75% reduction from day 0 to day 14; 95% from day 0 to day 28) than for MFAT LP (25% reduction from day 0 to day 14; 50% from day 0 to day 28) (Figure 3(a)).

A similar trend was observed by evaluating specifically the MSC contents in MFAT and in LP (Figure 3(b)): in MFAT, the MSC number was stable until day 14, and after 21 and 28 days of culture, the reduction was 50% and 75%, respectively; MSC number resulted significantly higher in MFAT than in LP (Figure 3(c)).

### 3.4. Long-Lasting Anti-Inflammatory Activity of MFAT-CM

**Figure 4:**
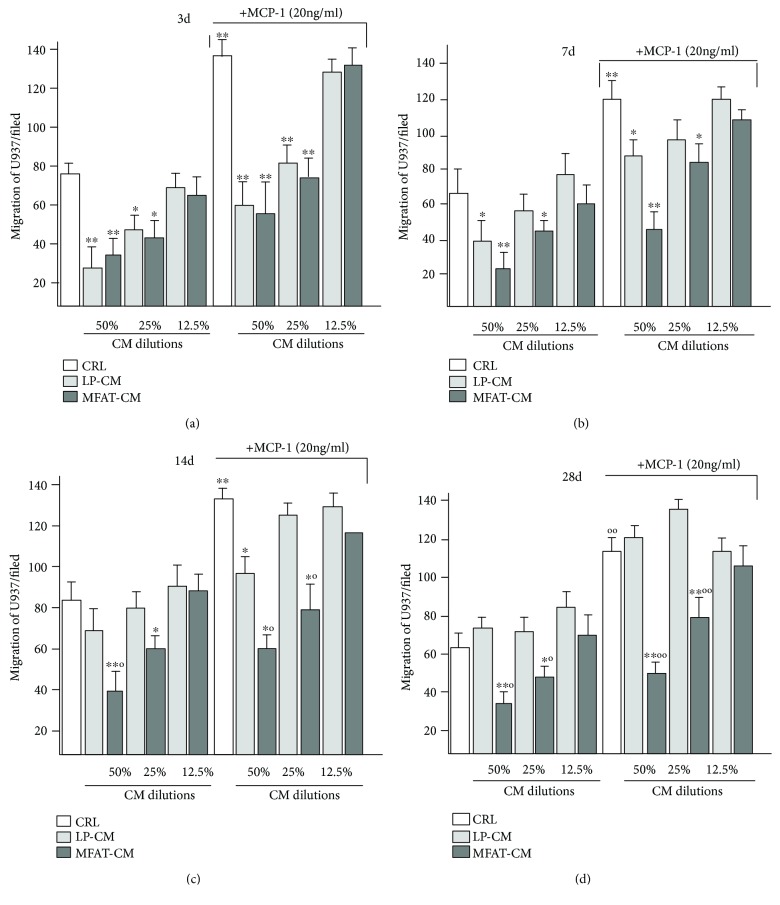
MFAT-CM induces a long-lasting block of U937 migration. CMs were recovered at 3, 7, 14, and 28 days from MFAT and LP specimens cultured in DMEM serum-free medium. Migration of U937 was evaluated by using Transwell inserts. CM was placed in the lower well at different dilutions. The cells placed in the upper well and migrated through a filter (5 *μ*m pore size) to the lower well were counted after 6 h. MFAT-CM inhibit the U937 migration also after long-term culture, whereas LP-CM was effective until 7 days. MCP-1 (20 ng/ml) was used as positive control to stimulate U937 migration. The bars represent the average ± SD of the total number of cells found in 10 different fields for each membrane at 40x magnification. ^∗^*p* < 0.05 and ^∗∗^*p* < 0.01 vs. CTRL; °*p* < 0.05 and °°*p* < 0.01 vs. LP-CM.

**Figure 5:**
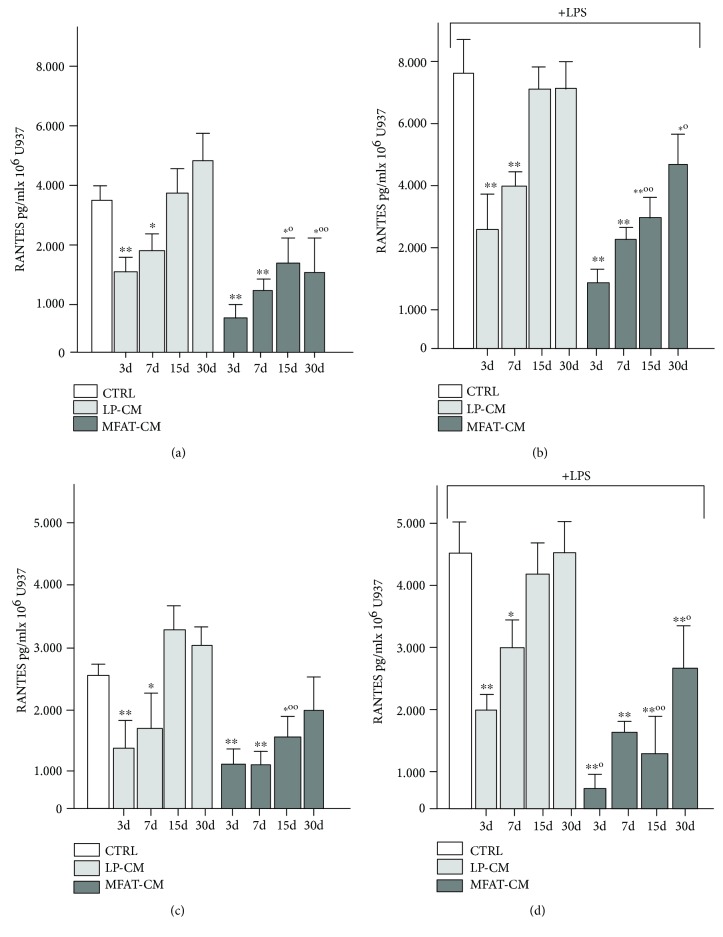
MFAT-CM inhibits RANTES and MCP-1 secretion by U937. CMs were recovered at 3, 7, 14, and 28 days from MFAT and LP specimens cultured in DMEM serum-free medium and added to U937cells at 1 : 2 dilution. The cells were incubated for 48 h and then medium was recovered to evaluate RANTES (a) and MCP-1 (b) concentration. Experiments were repeated in the presence of LPS (c, d) that stimulate secretion of both chemokines by U937. MFAT-CM resulted able to inhibit the secretion of RANTES and MCP-1 by U937 even in the presence of LPS (1 *μ*g/ml) stimuli. The bars represent the average ± SD of 5 different donors analyzed. ^∗^*p* < 0.05 and ^∗∗^*p* < 0.01 vs. CTRL; °*p* < 0.05 and °°*p* < 0.01.

We investigated the anti-inflammatory activity of MFAT-CM and LP-CM at early and late time of culture on the monocyte/macrophage U937 cell line, which has been used as model to investigate inflammation [24, 25]. We analyzed the ability of MFAT-CM and LP-CM to affect migration of U937 under basal condition or in the presence of MCP-1, a chemokine able to stimulate their motility (Figure 4) [26]. We found that both MFAT-CM and LP-CM recovered after 3 days of incubation, at both dilutions (12.5% and 50%), were able to inhibit U937 migration either in the presence or in the absence of MCP-1 stimuli (Figure 4(a)). At day 7 of incubation, the inhibitory activity of LP-CM was present only at higher concentration (50%), whereas MFAT-CM blocked migration even at lower dilution (Figure 4(b)). At days 14 and 28, LP-CM lost efficacy; in contrast, MFAT-CM continued to block U937 migration (Figures 4(a) and 4(d)). The anti-inflammatory activity of MFAT-CM and LP-CM was also investigated by evaluating the release of inflammatory cytokines RANTES and MCP-1 by U937 (Figure 5). The U937 cell line was cultured for 48 h in the presence of LP-CM or MFAT-CM. Similarly to those observed on migration experiments, the addition of both LP-CM and MFAT-CM (day 3 of culture), were very effective in reducing RANTES (Figure 5(a)) and MCP-1 (Figure 5(b)) secretion and the inhibition activity persisted also for CM recovered at day 7 of culture. At days 14 and 28 of culture, the inhibitory activity was absent for LP-CM, whereas it was still present for MFAT-CM (Figures 5(a) and 5(b)). The inhibitory activity of MFAT-CM and LP-CM was maintained also in the presence of LPS (Figures 5(c) and 5(d)).

## 4. Discussion

The use of adipose tissue has gradually developed into an exciting new way to be used in tissue regeneration: autologous fat grafting and the use of optimized SVF have become a hot topic with potential high-value clinical translation.

Commercial SVF preparation systems have previously highlighted the necessity for in-depth analysis of safety profiling, viable cell analysis, and ultimately clinical trials in order to understand and finely tune the action and benefit [27]. The “quality” of fractions and their MSC content may indeed vary from patient to patient [28]; however, so far, when used in various treatment regimens, these differences have not been shown to significantly alter the outcome [29]. SVF or their purified MSC content has been reported in the literature to have potential therapeutic value [30], but a full characterization of component vs. effect is still lacking. Fat-derived SVF and even more MSC preparations require a significant tissue manipulation with difficulties to meet the complex GMP guidelines required for their clinic applications [31].

For all these reasons, it is necessary to develop new technologies, GMP compliant, that minimized fat tissue manipulation. Recently, Tremolada and colleagues [16] developed a simple self-contained mechanical technique to produce a microfragmented adipose-derived fraction (MFAT). This enzyme-free technology is able to convert lipoaspirate (LP) into MFAT using a device named Lipogems® and leads to prepare MFAT that contains SVF and a significant number of MSCs [19] and ECs. Thanks to this procedure MFAT can be prepared intraoperatively in a very short time (15-20 min) [17].

Scope of this work was to investigate the long-term survival and content of MSCs and ECs as well the anti-inflammatory activity of LP and its derived MFAT, in vitro, with the aim to better understand their possible in vivo mechanism of action particularly when transplanted in inflammatory diseases [18, 32].

Here, we show that mechanical treatment of LP to obtain MFAT resulted in a significant increase of MSC survival with a prolonged secretory activity, in vitro, under serum-free condition, for more than 4 weeks.

Secretome analysis demonstrate that MFAT produced a significant higher level of G-CSF, SCGF-*β*, and HGF compared with LP. G-CSF production in MFAT could be associated to the activation of ECs probably due to the shearing force produced by the device. G-CSF has previously been shown to be a critical factor in augmenting tissue regeneration of cartilage repair [33], in dermal and epidermal wound healing [34], and rotator cuff healing and repair [35]. Moreover, G-CSF stimulates the production and activation of MSCs, induces increased expression of stem cell growth factor, HGF [36], and improved tissue recruitment capacity and anti-inflammatory status (inhibition of IL-10 and TNF-*α*) [37].

Further examination of viability and cell number indicated greater stability in MFAT in terms of total cell count and MSC content compared with LP. It was interesting to observe that in MFAT, after 28 days of culture, the initial number of MSCs was reduced to 70%, while the release of cytokines decreased to 30%. This data seems to suggest that, particularly in MFAT, a significant amount of cytokines initially secreted by the endothelium, MSCs, and by other cells (pericytes, fibroblasts) could remain entrapped in the extracellular matrix and is slowly released over the incubation time due of matrix degradation. In LP, matrix degradation could occur more rapidly and this may explain why at 7 days the secretome had a significant higher level of cytokines but this level decreases quickly over time.

This “stability” may be probably associated to the structural inner characteristic of MFAT: MFAT is composed by small aggregates of “homogeneous” size [19, 37] that naturally preserved their cell content, as a “bioreactor.” In fact, MFAT appears to be resistant to environmentally poor tissue culture conditions (serum free) and is capable of long-term cytokine release [38]. When the aggregates are disintegrated in syringe-based fat processing or using enzyme-based techniques, it results in a rapid loss in cell content and viability and cytokine secretion.

In this study, we further showed that MFAT-CM was able to inhibit U937 monocyte/macrophage migration even in the presence of MCP-1 and this ability was retained even after 28 days of culture; in addition, secretion of MCP-1 and RANTES was similarly and significantly reduced over the same period even in the presence of stimulating LPS. In contrast, LP-CM is less effective. Moreover, previous studies have shown that stem cells can stimulate MCP-1 and RANTES and this is led to inflammation through recruitment of blood leukocytes in a proinflammatory environment [39, 40]. Moreover, previous study on osteoarthritic-derived chondrocytes showed that inflammatory cytokine production (such as RANTES and MCP-1) could be modulated by adipose-derived stem cell contact but not by their derived conditioned medium [41]. This suggests that the potency of secretome derived from MFAT samples may be superior to those obtained by untreated LP or by purify MSCs.

It is well known that vascular injury is one of the main causes that determines “vascular activation” and consequently the release of angiogenic and growth factors, as well immune modulators and cytokines [42]. MFAT preparation is the result of LP transformation by a mechanical procedure that breaks up the fat tissue determining a great fragmentation of blood microvessels without affecting MSC and EC viability [19, 37]. In addition, Harting and colleagues have shown that the inflammatory stimulation of MSCs improved the anti-inflammatory activity of their secreted EVs [14]. Actually, we do not know whether the long-lasting anti-inflammatory activity of MFAT secretome can be directly related to EV content and if this process can be exacerbated by the vascular activation of MFAT specimens. However, it is known that, upon vascular injury, endothelial cells release EVs and molecules that play important roles in inflammation, angiogenesis, and thrombosis [43, 44]. We think that the potent anti-inflammatory activity of MFAT secretome is a very complex phenomenon that could probably depend to a combination of molecules and EVs released either by MSCs or the activated endothelium. Very recently, Carelli and colleagues showed that mechanical activation of fat tissue improves its anti-inflammatory properties [45]; therefore, it can be affirmed that the main differences between LP and MFAT here described are probably due to the different “grade” of fat tissue activation (more specifically endothelium activation?), clearly superior in MFAT than in LP.

LP has been shown to have therapeutic efficacy *in vivo* [46], and, when supplemented with SVF, resulted in better neovascularization and immunomodulation.

MFAT may be effective because it combines a natural structural scaffold organization with a very well preserved SVF and an extended MSC survival also in a hostile inflammatory microenvironment. To date, no comparative studies in human are available to demonstrate the major efficacy of MFAT vs. LP for inflammatory diseases. Our data seem to indicate mechanistic insights as to the benefits of MFAT preparations when compared with standardized fat aspirates for clinical use.

A key challenge in regenerative medicine is tissue minimal manipulation, and we hope that this study may open the door to possible optimization processes associated with fat grafting operative procedures [47].

## Figures and Tables

**Table 1:** Comparative analysis of MFAT and LP.

AT (1 ml)	Age	Gender	Protein (*μ*g/ml)	DNA (ng/ml)	Total cells (*n* × 10^5^/ml)	% CD31^pos^	% CD31^neg^	Total MSCs × 10^3^/ml	% MSCs CD31^neg^/ml
MFAT	**29-78**	**13F/4M**	**0.11-0.67** (**0.39**)	**34-103** (**65.5**)	**1.8-5.6** (**3.7**)	**54-86** (**72.7**)^∗^	**14-46** (**27.3**)	**3.7-12.4** (**7.2**)	**15-26.9** (**26.3**)^∗^
LP			**0.31-2.46** (**0.82**)^∗^	**64-128** (**83.5**)	**2.9-8.9** (**5.7**)^∗^	**40-67** (**50.5**)	**33-60** (**49.5**)^∗^	**6.6-12** (**9.8**)	**11-19.8** (**16.6**)

^∗^ *p* < 0.05; AT: adipose tissue; *n* = 17 donors analyzed.

**Table 2:** MFAT, LP, and MSC secretome at 72 h of incubation.

Analytes	MSCs-CM (*n* = 3)	MFAT-CM (*n* = 8)	LP-CM (*n* = 8)
IL-1r*α*	32 ± 118	39 ± 23	—
IL-6	1930 ± 658^	76 ± 47	26 ± 16
IL-8	545 ± 65	4290 ± 2431	4112 ± 1540
IL-12p70	149 ± 34	27 ± 14	17 ± 12
*β*-FGF	19 ± 12	808 ± 324^∗^	973 ± 321^∗^
G-CSF	178 ± 665	681 ± 437^∗^	23 ± 13
GM-CSF	87 ± 21	67 ± 13	65 ± 6
MCP-1	1437 ± 432^	105 ± 67	116 ± 56
PDGF-BB	—	31 ± 19	—
RANTES	78 ± 43	197 ± 36	164 ± 25
TNF-*α*	23 ± 2	33 ± 18	32 ± 15
VEGF	1409 ± 564^	302 ± 67	238 ± 65
IL-2r*α*	17 ± 4	32 ± 7	32 ± 3
IL-3	99 ± 34	77 ± 18	54 ± 9
IL-12p40	130 ± 35	196 ± 39	155 ± 32
IL-16	57 ± 46	612 ± 142^∗^	624 ± 110^∗^
IL-18	14 ± 2	—	39 ± 21
CTACK	30 ± 11	32 ± 24	54 ± 9
GROa	289 ± 78^	86 ± 56	65 ± 12
HGF	111 ± 112	4145 ± 755^∗^	2505 ± 451^∗^
LIF	36 ± 21	65 ± 24	—
M-CSF	31 ± 18	109 ± 93	43 ± 4
MIF	133 ± 34	8358 ± 2675^∗∗^	9234 ± 1121^∗∗^
MIG	14 ± 5	1055 ± 321^∗∗^	1326 ± 289^∗∗^
SCF	30 ± 23	74 ± 34	42 ± 3
SCGF-*β*	11283 ± 4490^^	1972 ± 903	477 ± 112
SDF-1	189 ± 27	71 ± 25	56 ± 11
TRAIL	15 ± 5	125 ± 57	111 ± 34

^*p* < 0.05; ^^*p* < 0.01 vs. LP-CM or MFAT-CM; ^∗^*p* < 0.05 vs. MSCs-CM. *n* = number of samples tested.

**Table 3:** MFAT and LP secretome at 3, 7, 14, and 28 days *in vitro*.

Analytes	3 days of culture		7 days of culture		14 days of culture		28 days of culture	
	MFAT-CM	LP-CM	MFAT-CM	LP-CM	MFAT-CM	LP-CM	MFAT-CM	LP-CM
IL-1r*α*	55 ± 18	53 ± 17	51 ± 27	151 ± 78	121 ± 43	—	48 ± 23	—
IL-6	65 ± 23	276 ± 67	403 ± 98	3982 ± 90^∗^	219 ± 87^∗^	7 ± 5	95 ± 31	3 ± 2
IL-8	3770 ± 1245	2860 ± 890	4777 ± 1769	8108 ± 1422^∗^	1038 ± 432	512 ± 102	463 ± 145^∗^	112 ± 46
IL-12p70	—	—	—	220 ± 88	114 ± 32	—	77 ± 14	—
*β*-FGF	650 ± 231	518 ± 198	302 ± 96	134 ± 67	130 ± 54	154 ± 56	66 ± 25	126 ± 85
G-CSF	398 ± 123	839 ± 234	73 ± 54	238 ± 98	180 ± 38	—	—	—
GM-CSF	79 ± 32	61 ± 34	61 ± 43	73 ± 13	62 ± 25	71 ± 34	60 ± 35	—
MCP-1	317 ± 145	317 ± 78	283 ± 98	383 ± 121	351 ± 87^∗^	—	249 ± 124^∗^	
PDGF-BB	47 ± 239	32 ± 16	—	—	—	—	—	—
RANTES	193 ± 87	49 ± 21	—	114 ± 33	—	—	—	—
VEGF	515 ± 145	251 ± 78	308 ± 30	1446 ± 799^∗^	749 ± 68^∗^	123 ± 87	479±39^∗∗^	63 ± 18
IL-2r*α*	—	—	—	—	52 ± 18	—	47 ± 26	—
IL-3	82 ± 43	66 ± 31	62 ± 15	81 ± 34	89 ± 25	—	75 ± 27−	
IL-12p40	224 ± 56	202 ± 50	170 ± 32	191 ± 87	202 ± 46	70 ± 31	201 ± 86^∗^	—
IL-16	332 ± 89	124 ± 43	130 ± 56	213 ± 111	58 ± 57	—	47 ± 15	—
GROa	68 ± 22	102 ± 42	134 ± 87	787 ± 234	58 ± 32	30 ± 15	41 ± 18	—
HGF	12003 ± 4657	7315 ± 2354	5490 ± 1677	5584 ± 1989	13442±3687^∗∗^	772 ± 232	9839±2341^∗∗^	239 ± 97
LIF	56 ± 16	56 ± 17	61 ± 23	177 ± 89	32 ± 13	—	17 ± 3	—
M-CSF	252 ± 69	116 ± 43	271 ± 99	354 ± 143	124 ± 65	—	60 ± 9	—
MIF	7912 ± 2368	10086 ± 2899	4565 ± 908	3382 ± 970	7915±768^∗∗^	1407 ± 398	6430±11387^∗∗^	127 ± 54
MIG	925 ± 329	1323 ± 675	1390 ± 455	4048 ± 1324	195 ± 76^∗^	—	51 ± 24	—
SCF	71 ± 45	114 ± 65	166 ± 77	205 ± 54	41 ± 32	—	22 ± 9	—
SCGF-*β*	5591 ± 1457	1343 ± 630	1203 ± 544	2492 ± 545	5699±1875^∗∗^	201 ± 54	4560±799^∗∗^	—
SDF-1	72 ± 21	82 ± 29	71 ± 32	96 ± 29	106 ± 51	62 ± 32	99 ± 30	—
TRAIL	85 ± 47	46 ± 12	37 ± 11	57 ± 21	41 ± 10	—	—	—
Total	33, 759 ± 6567	26, 279 ± 5688	20, 039 ± 4387	32, 872 ± 9854^∗^	31, 059±8975^∗∗^	3, 329 ± 1254	23, 057±6590^∗∗^	706 ± 154

^∗^ *p* < 0.05; ^∗∗^*p* < 0.01 MFAT-CM vs. LP-CM.

## Data Availability

All the data (microscopy images, ELISA test, cell count, FACS analysis, and so on) used to support the findings of this study are available from the corresponding author upon request.
